# Phloem small RNAs, nutrient stress responses, and systemic mobility

**DOI:** 10.1186/1471-2229-10-64

**Published:** 2010-04-13

**Authors:** Anja Buhtz, Janin Pieritz, Franziska Springer, Julia Kehr

**Affiliations:** 1 Centro de Biotecnología y Genómica de Plantas (UPM-INIA), Campus de Montegancedo, M40 (km38), 28223 Pozuelo de Alarcón/Madrid, Spain; 2 Max Planck Institute of Molecular Plant Physiology, Department Lothar Willmitzer, 14476 Potsdam, Germany

## Abstract

**Background:**

Nutrient availabilities and needs have to be tightly coordinated between organs to ensure a balance between uptake and consumption for metabolism, growth, and defense reactions. Since plants often have to grow in environments with sub-optimal nutrient availability, a fine tuning is vital. To achieve this, information has to flow cell-to-cell and over long-distance via xylem and phloem. Recently, specific miRNAs emerged as a new type of regulating molecules during stress and nutrient deficiency responses, and miR399 was suggested to be a phloem-mobile long-distance signal involved in the phosphate starvation response.

**Results:**

We used miRNA microarrays containing all known plant miRNAs and a set of unknown small (s) RNAs earlier cloned from *Brassica *phloem sap [1], to comprehensively analyze the phloem response to nutrient deficiency by removing sulfate, copper or iron, respectively, from the growth medium. We show that phloem sap contains a specific set of sRNAs that is distinct from leaves and roots, and that the phloem also responds specifically to stress. Upon S and Cu deficiencies phloem sap reacts with an increase of the same miRNAs that were earlier characterized in other tissues, while no clear positive response to -Fe was observed. However, -Fe led to a reduction of Cu- and P-responsive miRNAs. We further demonstrate that under nutrient starvation miR399 and miR395 can be translocated through graft unions from wild type scions to rootstocks of the miRNA processing *hen1-1 *mutant. In contrast, miR171 was not transported. Translocation of miR395 led to a down-regulation of one of its targets in rootstocks, suggesting that this transport is of functional relevance, and that miR395, in addition to the well characterized miR399, could potentially act as a long-distance information transmitter.

**Conclusions:**

Phloem sap contains a specific set of sRNAs, of which some specifically accumulate in response to nutrient deprivation. From the observation that miR395 and miR399 are phloem-mobile in grafting experiments we conclude that translocatable miRNAs might be candidates for information-transmitting molecules, but that grafting experiments alone are not sufficient to convincingly assign a signaling function.

## Background

The levels of essential inorganic nutrients have to be tightly controlled inside individual cells and organs, but information about nutrient uptake and needs also have to be transferred between organs to optimize nutrient allocation, especially in plants growing under sub-optimal conditions. If an organ experiences nutrient starvation, it needs to communicate its requirements to the other organs in order to increase nutrient uptake or reallocate resources. This type of communication is probably mediated via the phloem. Recent work showed that microRNA (miRNA) 399 is potentially involved in long-distance communication via the phloem following phosphate deprivation [1-3]. miRNAs are short (21-24 nt), non-translated RNAs that are processed by Dicer-like proteins from large, characteristically folded precursor molecules. The majority of plant miRNAs target transcription factors and is therefore thought to mainly regulate developmental processes. However, recent studies have also identified miRNAs that are involved in responses to nutrient deficiencies. As mentioned earlier, miR399 is strongly induced during phosphate deprivation [4-7], while miR395 drastically increases under growth on low sulfur [8]. In addition to macronutrients like sulfur and phosphate, also a lack of the micronutrient copper leads to an accumulation of miR397, 398, 408, and 857 [9-11]. miRNAs 395, 398 and 399 were recently shown to accumulate not only on the whole plant level, but also strongly within the phloem [1]. Since sRNAs accumulating in phloem sap under stress could represent potential long-distance signaling molecules, we used sRNA microarrays from LC Sciences to comprehensively analyze phloem sRNAs. The customized arrays contained, in addition to all known plant miRNAs, a subset of small RNAs (sRNAs) of unknown function that was earlier sequenced from phloem sap of *Brassica napus *[1]. First we established the miRNA patterns of phloem, leaves and roots of fully nutrient supplied, hydroponically grown oilseed rape plants to subsequently identify candidates that respond to growth under S, Cu or Fe deficiency, respectively. In addition, we used the highly -S induced miR395 as an example to examine whether this specific miRNA can be transported over graft unions when combining WT Arabidopsis with the miRNA biosynthesis mutant *hen1-1*. The specific aims were 1) to find phloem- and organ-enriched miRNAs, 2) to identify additional miRNAs that respond to S and Cu deficiencies, 3) to examine whether any miRNAs respond to Fe starvation, and 4) to demonstrate whether miR395 is phloem mobile or not.

## Results and Discussion

### Phloem sap shows a specific sRNA pattern that is distinct from that of inflorescence stem, leaves and roots

To ensure that the sRNAs observed in phloem sap were not resulting from contamination during sampling, and in order to identify phloem-enriched sRNAs, we performed a microarray hybridization experiment comparing phloem sap to the surrounding inflorescence stem tissue. This resulted in the identification of phloem-enriched sRNAs, while others were less abundant in phloem sap than in stem tissue (including phloem) collected after phloem sampling from the sampling site. Signal values for one miRNA per family are depicted in additional file 1. The distribution of ten miRNAs was re-evaluated by RNA gel blots from an independent set of plants, what confirmed the microarray results. miRNAs 162, 167, 168, 169, and 399 strongly accumulated in phloem samples as compared to inflorescence stem samples, while miR158, 396 and 397 were stem-enriched. This indicates that phloem samples are not significantly contaminated by the contents of the surrounding inflorescence stem cells, what had already previously been demonstrated [1,12]. The observation that miR167 accumulates in phloem sap confirms an earlier study in pumpkin that found miR167 20-fold enriched in phloem sap as compared to the surrounding vascular tissue [13]. Also the failure to detect miR171 in phloem sap and its low expression in stem samples is in accordance with earlier findings [13,14].

**Figure 1:**
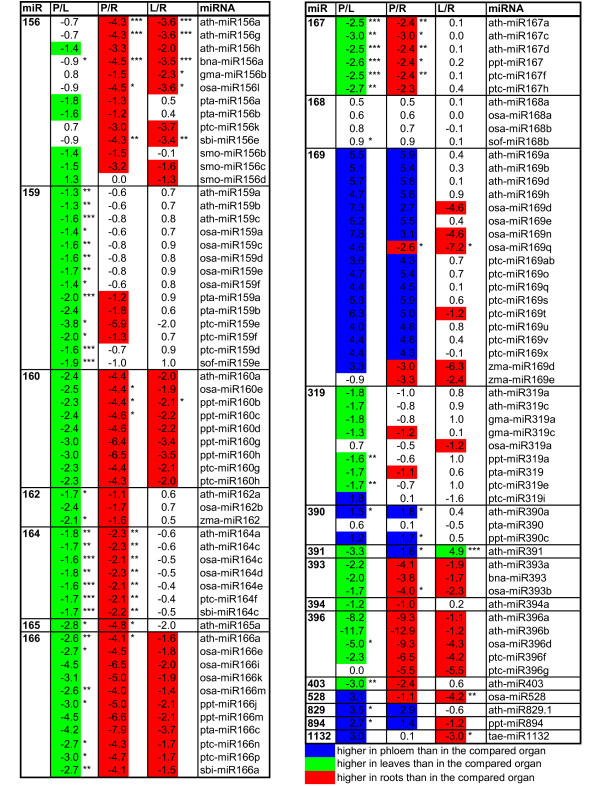
**List of miRNAs that were enriched in phloem, leaves or roots, respectively, in plants grown under full nutrition**. Only families where at least one member showed a statistically significant differential accumulation in one organ are shown (p < 0.05, n = 3). Values are log2s between P/L: phloem vs. leaves, P/R: phloem vs. roots and L/R: leaves vs. roots. Markedly (log2 values >1 or <-1, indicating a two-fold difference) phloem-enriched miRNAs are marked in blue, leaf-enriched in green, and root-enriched in red. The statistical significance is indicated as: * p < 0.05; ** p < 0.01; *** p < 0.001.

We further used the microarrays to identify sRNAs that preferentially accumulated in phloem sap as compared to leaf and root samples. To this end we grew plants under full nutrition (FN) conditions in three successive, completely independent experiments and compared the sRNA amounts in phloem samples with that of leaves and roots. For inter-array comparisons, signal intensities were normalized to the median signal of each sample. This approach allowed the detection (signal >100) of 161 miRNAs belonging to 37 families in phloem sap, covering all 17 miRNA families earlier detected in samples from soil-grown *Brassica *plants by high-throughput pyrosequencing [1] (indicated by the numbers of sequences obtained in additional file 1). In addition, we found several miRNAs on the arrays that were not identified by the sequencing approach, suggesting that these miRNAs were either not present in soil-grown plants or not identified, possibly due to their low abundance or absence in the steadily growing databases at the earlier time-point of data analysis. A reasonable reproducibility between the experiments was achieved, given that they were completely independent and that miRNAs are known to be strongly influenced by developmental stage and growth conditions [15]. Signal intensities and standard deviations for one representative of each family are depicted in additional file 2. Statistical evaluation using the Students t-test revealed miRNAs that were significantly (p < 0.05) enriched in phloem, leaves or roots (figure 1). miRNAs from four families were more abundant in phloem sap than in leaves and roots under FN, namely miR169 (not statistically significant), 390, 829, 894, and 1132 (not significant) (figure 1). miR1132, together with miR1134 (misnamed miR518), was cloned from wheat [16] and recently from *Brachypodium *[17]. Both miRNAs are not well characterized, thought to be species-specific, and their possible functions are unknown. However, signal values were well above the microarray noise. Nevertheless this result does not allow a conclusion on whether these miRNAs really occur in *Brassica *or if the signals represent an artifact (e.g. unspecific cross-hybridization) caused by the microarray technique.

Except for miR390, these miRNAs were also phloem-enriched as compared to inflorescence stem tissue (additional file 1). miRNAs from the families 156, 159, 160, 162, 164, 165, 166, 167, 393, 394, 396 and 403 were less abundant in the phloem as compared to both, leaves and roots. However, some of these miRNAs (159, 162, and 167) were more abundant in the phloem than in the surrounding stem.

miRNAs from the complete 156, 160, 166, 393, 396, and 528 families were found to be significantly enriched in roots as compared to leaves and phloem. In rice, miRNAs 156 and 166 have earlier been shown occur at higher levels in roots than in leaves [18]. In addition, miR166 has been described to be expressed in roots of *Medicago truncatula*, where it functions in root and nodule development [19]. In Arabidopsis, miRNAs 156 and 160 occur root-enriched [20], and miR160 has been implicated with root development [21,22].

miR391 was the only miRNA that accumulated in leaves as compared to roots and phloem sap (figure 1). In an earlier study, miR391 was found to appear preferentially in rosette leaves of Arabidopsis, as compared to seedlings, flowers and siliques [23]. According to the same publication, miR391 targets a beta-fructofuranosidase, but its function is currently not well understood. Although miR391 is regarded as being related to miR390, differing in only 5 nt [24], both miRNAs showed a quite distinct organ distribution: while miR391 was clearly leaf-enriched, miR390 was slightly, but significantly phloem-enriched, indicating that both miRNAs might still have distinct localizations and functions.

**Figure 2:**
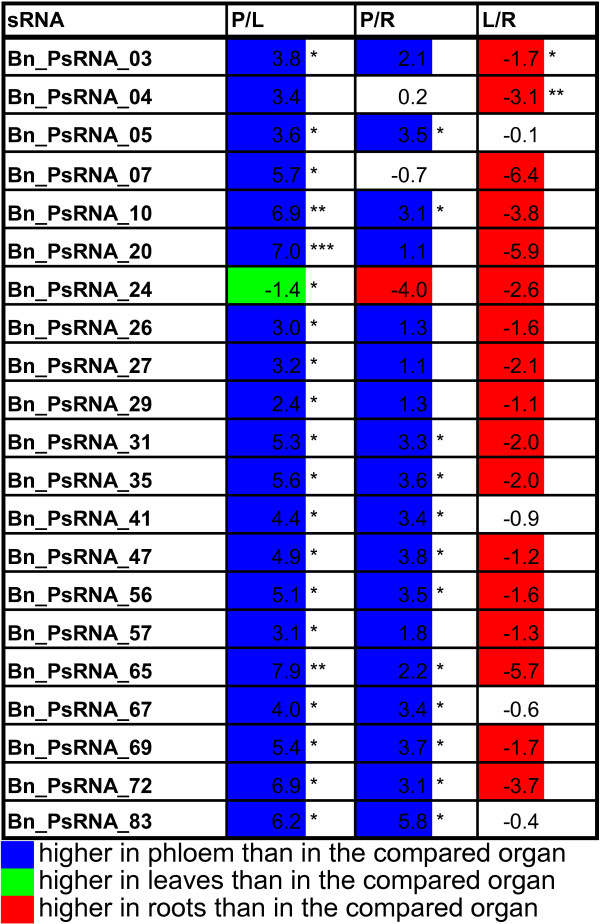
**List of unknown sRNAs that were organ-enriched grown under full nutrition**. List of unknown sRNAs, sequenced from *Brassica *phloem sap [1], that showed statistically significant differences between phloem sap, leaves and roots, respectively (p < 0.05, n = 3). Values are log2s between P/L: phloem vs. leaves, P/R: phloem vs. roots and L/R: leaves vs. roots. Markedly (log2 values >1 or <-1, indicating a two-fold difference) phloem-enriched miRNAs are marked in blue, leaf-enriched in green, and root-enriched in red. The statistical significance is indicated as: * p < 0.05; ** p < 0.01; *** p < 0.001.

Interestingly, the unknown sRNAs represented on the chip were, except for Bn_PsRNA_24, significantly more abundant in phloem sap as compared to leaves and roots (figure 2). All Bn_PsRNAs were additionally more abundant in roots than in leaves. Most of these differential unknown sRNAs had a length of 24 nt, and only five had a length of 21 nt characteristic for miRNAs (figure 2). Precursor and target predictions using mfold and psRNATarget, respectively (data not shown), provided no conclusive evidence that any of these sRNAs could represent a novel miRNA following recently published criteria [25]. On the one hand, the inability to successfully predict targets and precursors of the Brassica sRNAs could be due to the limited EST genome sequence of *Brassica napus *publicly available. On the other hand, it could indicate that they are no miRNAs, but rather siRNAs, as yet unclassified sRNAs, or breakdown products of larger RNAs. However, the observation that they accumulate in phloem sap makes them interesting candidates for future studies.

### Phloem small RNA patterns change under nutrient deficiency

Since three miRNAs, miR395, 398 and 399, had been previously shown to accumulate in the phloem under the corresponding nutrient stress conditions [1], we intended to identify additional nutrient-responsive phloem sRNAs. They could represent novel information transmitters during nutrient deprivation, as has been suggested for miR399 under phosphate deficiency [2]. To induce nutrient deprivation, we raised *Brassica napus *plants in hydroponic cultures under FN and omitted the respective nutrient from the medium for two (-S, -Cu experiments) or three weeks (-Fe experiment), respectively, before samples were collected. Under -S and -Cu conditions the plants did not show any obvious stress symptoms at the time of sampling. However, omitting Fe led to chlorosis symptoms in very young upper leaves after 4-5 days of stress (data not shown).

Initial analysis of the expression of selected genes that are known to be altered by the respective nutrient stress clearly confirmed that the plants were nutrient deficient in all three kinds of stress experiments performed (additional file 3). As expected, S starvation led to an increase in the expression of the two high-affinity sulfate transporters st1 (AJ416460) and st2 (AJ311388), especially in roots. Copper deprivation was confirmed by a slight decrease in the amount of Cu-Zn SOD transcripts, while the amount of the high-affinity copper transporter COPT1 increased markedly. Fe deprived plants showed only a slight reduction in the expression of the iron storage protein ferritin LSC30 in leaves and roots, accompanied by an increase in the transcript of the root-specific iron transporter IRT1 in roots (additional file 3).

Subsequently, material from the same batch of plants was used for dual-color microarray hybridizations of stressed and FN samples. Since only one array per stress experiment was hybridized, we applied specific criteria to only identify the most drastic positive changes (>four-fold increases, log2 >2) upon stress treatments and furthermore restricted the analyses to abundant sRNAs with signal intensities of >100 in one of the two (FN or stressed) samples.

**Figure 3:**
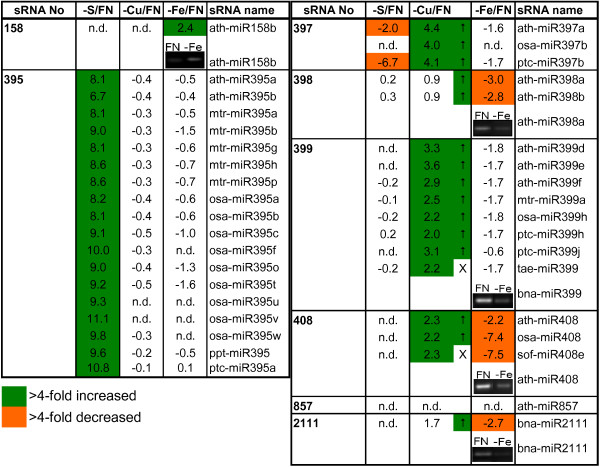
**List of nutrient-responsive sRNAs**. List of sRNAs that showed a strong positive reaction to S, Cu or Fe deprivation, respectively, shown as log2 values of stressed vs. FN samples. Only sRNAs that fulfilled the criteria described in the Methods section (positive response, log2 >2 in one of the stress treatments, signal value >100 in FN or deprived sample) in at least one of the comparisons are listed. The insets show results obtained by miRNA sqRT-PCR (after 25 cycles) from an independent experiment. To allow a better overview, values for known nutrient starvation-responsive miRNAs (398 and 857 for -Cu and 2111 for -P) were included, although they only showed a negative response or were not detectable. Arrows indicate directions of changes obtained in a second, independent -Cu experiment. n.d.: not detectable (both, FN and stress, signal values <100). X: not on chip.

The response to S deficiency was characterized by a dramatic increase of the known -S-responsive miR395 (the at-miR395a signal increased from 280 to 76369). While the amount of no additional miRNA increased, the amount of miR397 decreased (figure 3).

**Figure 4:**
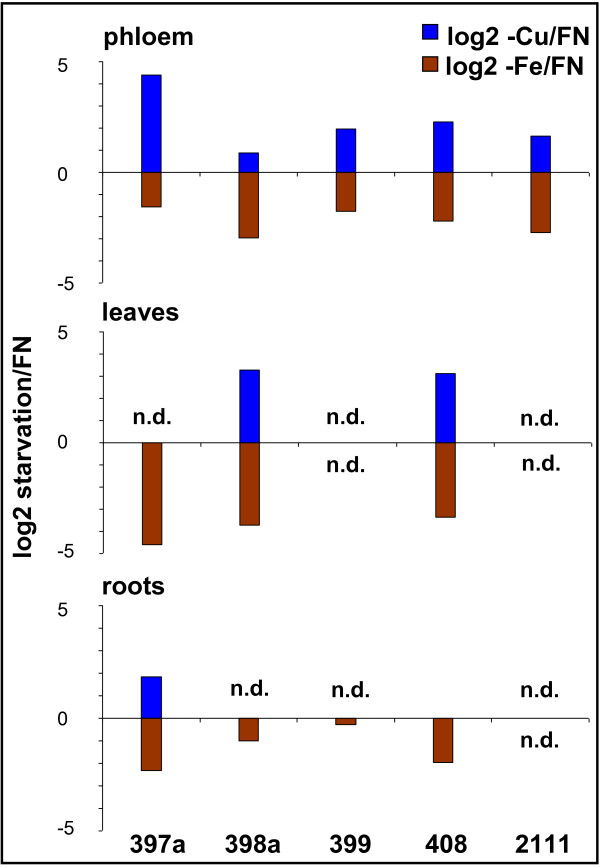
**Effect of copper and iron deficiency on known nutrient-responsive miRNAs**. Graphic summary of the opposite effect of copper and iron deficiency on the known -Cu responsive miRNAs 397, 398, 408 and the -P responsive miRNAs 399 and 2111. Phloem responses are compared to data obtained from leaves and roots. All data were obtained from miRNA array hybridization experiments. Differences between stress and control plants are shown as log2 values, only Arabidopsis miRNAs are depicted. n.d.: not detectable.

Growth under copper deficiency is known to induce a number of physiological responses, including the expression of specific miRNAs. Recently, the transcription factor SPL7 (SQUAMOSA promoter binding protein-like7) has been found to be a central regulator of the copper-deficiency response. It is able to induce the expression of miRNAs 397, 398, 408, 857, different copper transporters, and a copper chaperone [26]. Accordingly, our miRNA microarrays showed that copper deficiency led to a more than four-fold increase of the known copper-responsive miRNAs 397 and 408 that target laccases [1,11] in phloem sap. miR397 also accumulated in roots, but remained undetectable in leaves, while 408 responded positively in leaves and not in roots (figure 4). The known -Cu-responsive miR398 that targets Cu/Zn superoxide dismutases also increased, but only nearly two-fold. A similar accumulation was also detected in leaves, but not roots (figure 4). miR857 that was found to be copper-responsive in Arabidopsis [11] was undetectable in the phloem, leaves and roots of rapeseed in the present study (figure 3), probably caused by the different species, compartment, developmental stage and milder stress treatment analyzed. Surprisingly, also the phosphate-deficiency-responsive miR399 increased more than four-fold (figure 3). This indicates a slight phosphate limitation in the -Cu plants, although the plants were supplied with the same amount of P as in all other experiments. The same was also observed in an independent repetition of the experiment (indicated by arrows in figure 3). Interestingly, miR2111 that was recently found to also respond to phosphate starvation [14] was also accumulating under -Cu, confirming the noticeable phosphate deficiency already evidenced by the increase of miR399 (figure 3). Our results thus confirm that copper deficiency up-regulates miRNAs that mainly target mRNAs of enzymes that use copper as cofactors, namely the multicopper proteins laccases and copper zinc superoxide dismutases (Cu/Zn SOD). As already discussed by Abdel-Ghany and Pilon [11], this mechanism is thought to save Cu for the most important copper-containing proteins like plastocyanin that is a key protein of photosynthesis [11].

Under iron deficiency only miR158 increased in the phloem more than four-fold (ath-miR158a increased from 231 to 1201), what was verified by sqRT-PCR in an independent experiment (inset in figure 3). miR158 was described as a non-conserved miRNA from Arabidopsis that could, for example, not be detected in citrus [27]. miR158 is predicted to target a pentatricopeptide repeat-containing protein of unknown function, a lipase, and xyloglucan-fucosyl transferases [28]. None of these potential targets has an obvious connection to iron uptake or metabolism, and thus the increase of miR158 might be a secondary effect on plant development. Moreover, the accumulation of miR158 seemed to be phloem sap-specific, as it could not be observed in leaf or root samples (see data submitted to GEO, series accession number GSE20263). Comparative high-throughput sequencing of FN and -Fe samples would help to clarify if an as yet unknown (and therefore not represented on the chip) sRNA increases under -Fe, or if there is really no small RNA accumulating during this deprivation response.

Interestingly, however, miRNAs 397, 398, 399, 408 and 2111 notably decreased during iron starvation, showing an opposite response to their increases observed under -Cu (figure 3, figure 4). This response was verified for miR398, 399, 408 and 2111 by sqRT-PCR from a set of independently grown plants (inset in figure 3). Decreases in the levels of -Cu-responsive miRNAs were visible not only in the phloem, but also in leaves and comparably weak in roots (figure 4). A decrease of these Cu starvation-responsive miRNAs suggests that copper uptake is stimulated by iron deficiency, as has already been observed in *Brassica *and other plant species [29,30]. The need for higher Cu uptake under -Fe could be explained by the fact that many iron and copper-containing enzymes can substitute for each other when one of the two elements is present at suboptimal levels, e.g. SODs, cytochrome oxidase, or diiron oxidase [31,32].

Interestingly, a phloem response opposite to the -Cu reaction under -Fe was also observed for the -P-responsive miRNAs 399 and 2111, which were more than two- (399), respectively more than four-fold (2111) decreased. The responses of miR399 and miR2111 were undetectable in leaves and roots (figure 4). This confirms the observation from a previous study that demonstrated that miR399 responds stronger to -P in phloem sap than in leaves and roots [2]. The decrease of -P-responsive miRNAs in phloem sap suggests that Fe deficiency positively influences P uptake and metabolism, what has already been demonstrated in earlier studies e.g. [33,34]. The other way around, high Fe can lead to lower P concentrations in the plant [34]. If more Fe is taken up during growth under -Cu in order to replace Fe in Cu-containing enzymes, this could explain the observed increase of the -P-responsive miRNAs in phloem sap under Cu deprivation.

Taken together, the data from the -Cu and -Fe experiments indicate a tight link between iron and phosphate metabolism that has earlier been described. Moreover, they suggest a close linkage between iron and copper uptake, although it is known that in higher plants this link is at least not as close as, for example, in yeast or *Chlamydomonas*, where iron uptake is directly Cu-dependent [35,36]. It is interesting to note that the tissues/compartments analyzed react differentially to specific stress triggers, but the physiological meaning of this observation needs to be evaluated in future experiments.

### Specific miRNAs that accumulate in phloem sap under stress are also mobile in grafting experiments

**Figure 5:**
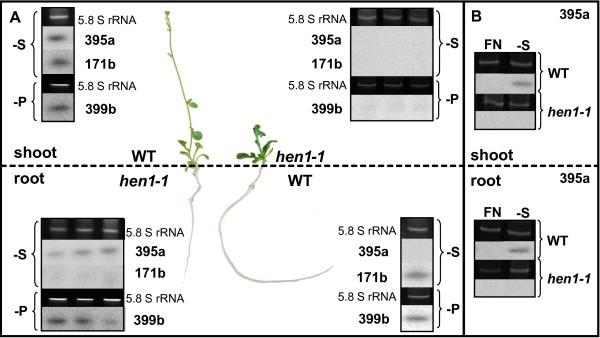
**WT/*hen1-1 *grafting experiments**. Analysis of mature miR395, miR399 and miR171 by RNA gel blot analysis in scions and rootstocks of reciprocal *hen1-1*/WT and WT/*hen1-1 *grafts under sulfate and phosphate deficiency. **A**: miRNAs 395 and 399 were translocated from WT scions to *hen1-1 *rootstocks but not in the opposite direction, miR171 was immobile. One representative result is shown for WT, and three replications for *hen1-1 *roots and shoots. The *hen1-1 *graft parts kept their growth retardation phenotype, indicating that not all necessary miRNAs could be transferred. The 5.8 ribosomal RNA band served as a loading control. **B**: Control of miR395 expression in WT and *hen1-1 *mutant plants. In WT plants miR395 was induced by sulfate deficiency in shoots and roots, while no signal was detected in *hen1-1 *mutants under both conditions.

Whether miRNAs are mobile between cells and over long distance is still a matter of debate and evidence for transport only exists for one single miRNA, miR399, that was able to move from shoots to roots in a miR399 overexpressor as scion/WT as rootstock graft situation [2,3]. Because miR395 is comparably well studied, its targets have been validated in Arabidopsis, and it strongly accumulates under sulfur starvation, also within the phloem, we chose this miRNA to examine whether additional miRNAs are mobile *in vivo*. To this end, we performed grafting experiments using *hen1-1 *mutants and WT plants. *hen1-1 *mutants are inhibited in sRNA methylation and, as a consequence, the levels of several miRNAs are markedly decreased [37]. RNA gel blot analysis of the different miRNAs further analyzed in our study confirmed that *hen1-1 *mutants did not contain any of these mature miRNAs at detectable levels (data not shown). In all grafting experiments, *hen1-1 *mutants retained their typical phenotype, mainly characterized by growth retardation (figure 5A), what indicates that not all necessary miRNAs can be translocated between the grafting partners. After the establishment of graft unions, successful grafts were transferred to media lacking a specific nutrient for two weeks, and miRNA abundance was analyzed in the different parts of the graft by RNA gel blots. We first examined the abundance of the phosphate-dependent miR399 in scions and rootstocks under phosphate starvation as a positive control. As expected, miR399 was not only clearly detectable in WT rootstocks and scions, but also in *hen1-1 *rootstocks of independent grafts with similar signal strength as in phosphate starved WT rootstocks (figure 5A). Our data thus confirmed the translocatability of miR399 from shoots to roots in a graft situation. We further chose miR171 as a negative control, since this miRNA has neither been detected in phloem sap by sRNA sequencing [1,14,38] nor by our sRNA array experiments (additional file 1). As assumed, we detected a signal in the WT rootstocks and scions, but not in the mutant parts of the grafts, making a phloem translocation of miR171 highly unlikely (figure 5A).

When analyzing grafts grown under sulfate starvation, we observed the translocation of miR395 from WT scions to *hen1-1 *rootstocks in different independently grafted plants. We also observed signals for miR395 in WT scions, but not in WT rootstocks (figure 5A). However, miR395 has been previously shown to be expressed in roots under sulfur starvation [39], and we could also detect signals in roots of intact WT plants (figure 5B). This result could be reproduced in several independent experiments. This could indicate that miR395 translocation from shoot to root is required for root miR395 expression in the WT, but further experiments will be needed to substantiate this assumption. The earlier studies of miR399 translocation do not allow any conclusions about the (non) existence of such a crosstalk, since a comparable graft situation of a stressed WT rootstock with an "unstressed" (not miRNA-producing) scion cannot be achieved when grafting overexpressors with WT plants [2,3].

For both, miR399 and miR395, we only found signals in *hen1-1 *rootstocks and never in *hen1-1 *scions, indicating that mobility was restricted to the direction from shoot-to-root in Arabidopsis seedlings (figure 5A). The reason for this unidirectional translocation might lie in the early developmental stage analyzed, where roots constitute the only real sink organ that needs nutrient supply from the phloem translocation stream. However, the results do not rule out that mobile miRNAs can reach other organs than roots at different developmental stages with different source-sink relationships. Our experiments also did not allow concluding whether mature miR395 or its PT is the translocated species. In the case of miR399, however, it has been previously shown that exclusively mature miRNA and not PTs is transported through graft unions [2]. In addition, no miRNA precursors were detectable in *B. napus *phloem sap [1], suggesting that mature miRNAs are the translocated molecules.

### The graft translocation of miR395 coincides with a down-regulation of the target APS4

**Figure 6:**
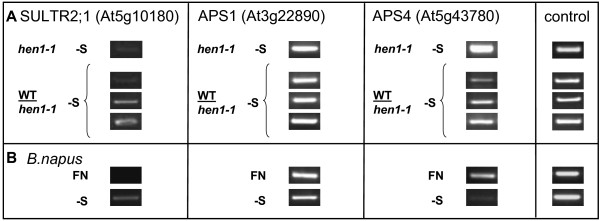
**Analysis of the targets of miR395 in roots**. Analysis of the mRNA levels of the miR395 targets SULTR2;1, APS1 and APS4 by semi-quantitative RT-PCR. **A**: PCR results from root tissue of hydroponically grown Arabidopsis *hen1-1 *mutants and WT/*hen1-1 *rootstocks (35 cycles, UBC10, At5g53300 served as a control). **B**: Changes of target mRNAs in *B. napus *roots under -S compared to full nutrition (FN) (35 cycles, UBP1B, At1g17370 served as a control).

To examine whether the translocation of miR395 from WT shoots into *hen1-1 *roots might have physiological functions, we analyzed the levels of three experimentally validated mRNA targets of miR395, the ATP sulfurylases *APS1 *and *APS4 *and the low affinity sulfate transporter *AtSULTR2;1 *[8,39]. As a general observation, the transcript levels of all three targets seemed to be higher in shoots of *hen1-1 *as compared to WT plants (additional file 4). In addition, the experiments showed that only the level of ATP sulfurylase *APS4 *mRNA, but not of *APS1 *or the low affinity sulfate transporter *SULTR2;1*, was notably decreased in grafted *hen1-1 *rootstocks as compared to non-grafted -S starved roots of *hen1-1*, while housekeeping genes remained constant (figure 6A). A similar reduction of levels of *APS4*, but not the other two targets, could be observed in *B. napus *WT roots grown under sulfur starvation (figure 6B). These results indicate that *APS4 *mRNA might be a target of miR395 in roots, and interestingly, this mRNA has previously been shown to exhibit root-specific expression [40]. The observation that the other miR395 target *SULTR2;1 *was up- and not down-regulated under -S conditions (figure 6A and 6B, [39]) was earlier explained by the spatially differential expression of *SULTR2;1 *and miR395 in xylem parenchyma and companion cells, respectively [39]. It was suggested that one of the major functions of miR395 was the down-regulation of *SULTR2;1 *expression in the phloem to restrict *SULTR2;1 *expression exclusively to the xylem [39].

### Is the transport of specific miRNAs of biological relevance in intact plants?

Most miRNAs are believed to act in a locally restricted manner, in contrast to the mobile class of siRNAs [41]. Their limited mobility is suggested by the closely correlating patterns of miRNA transcription and activity [42], the spatial restriction of miRNA gene expression [43,44], and the limited area of mature miRNA localization [45]. However, phloem mobility of miR399 across graft unions has been demonstrated in earlier studies by grafting miR399 overexpressor with WT plants [2,3]. In this study, we observed the transport of miR395 and 399 from WT scions to *hen1-1 *mutant rootstocks. Moreover, one of the miR395 targets, APS4, was down-regulated in grafted mutant roots. This indicates that miR395, like miR399, is transported from shoot to root to down-regulate its target(s). However, the question whether such a miRNA transport is physiologically relevant remains, since members of the miR395 and 399 families can indeed be synthesized in roots of wild type plants under the respective stress [7,39] (figure 5B). Interestingly, expression of miRNAs 395 and 399 was shown to be highly overlapping, being predominant in vascular tissue, especially in root phloem companion cells (CC) [7,39].

Different scenarios could explain the observation that specific miRNAs are present in phloem sap and mobile in grafting experiments: 1) None of the phloem miRNAs is specifically targeted for translocation, but instead a portion of all miRNAs highly expressed in CC leaks into sieve elements. No miRNA would represent a signaling molecule. 2) A portion of all miRNAs highly expressed in CC reaches phloem sap, but some of these miRNAs can act as long-distance regulators under certain physiological conditions. 3) Selected miRNAs synthesized in CC are specifically targeted for transport and only these are released into the phloem stream. In this case, all miRNAs present in the phloem would be translocatable information transmitters.

No matter how miRNAs reach phloem sap, they would then be swept away from source to sink organs (in our system from shoots to roots). The translocated miRNAs would probably exit the translocation stream into sink CC in an unspecific manner, as rather unselective unloading of macromolecules into sink tissues has been suggested [46]. Here, they would down-regulate their target mRNAs, no matter whether they are intended to function as signaling molecules or not.

If certain miRNAs should indeed be translocated to transmit information, one possible rationale could be that roots are unable to synthesize sufficient amounts of these miRNAs under stress, or that they need a trigger from the shoot to initialize miRNA synthesis. This might be suggested by the absence of mature miR395 in WT rootstocks of grafted plants that was, however, well detectable in roots of complete WT plants (figure 5). Another explanation might be that some organs experience nutrient deprivation earlier than others, and that the translocated miRNAs serve to coordinate physiological responses with plant parts that are not yet stressed and therefore do not yet synthesize stress-responsive miRNAs themselves. This would resemble the situation in grafted plants, where only scions of the graft produced the stress-induced miRNAs (stressed WT in this study, overexpressors in [2]), while rootstocks did not (*hen1-1 *mutants in this study, non-stressed WT in [2]).

## Conclusions

This study demonstrates that the phloem sap sRNA complement is distinct from that of stems, leaves and roots, and that a set of phloem-enriched sRNAs exists. It also shows that the abundance of several phloem sap sRNAs changes under nutrient deficiency conditions. While the results confirmed that the known miRNAs reacting to -S or -Cu, respectively, also respond in phloem sap, they provided no clear indications that the response to -Fe involves miRNA regulation, despite of influencing copper uptake/metabolism.

Grafting studies between WT plants and *hen1-1 *mutants demonstrated that two phloem stress-reactive miRNAs, 395 and 399, can indeed be transported from shoot to root in Arabidopsis seedlings, and that this translocation leads to a reduction of the amount of their target mRNAs in roots. The grafting experiments also revealed that not all miRNAs are phloem translocatable, since miR171 did not move.

Therefore, this study demonstrates that identifying phloem-enriched macromolecules and analyzing their translocation in grafting studies is a very useful approach to distinguish between phloem translocatable and non-mobile molecules. It is tempting to classify miR395 and 399 as systemic signaling molecules, because they not only move from source to sink, but also induce a measurable effect on their target mRNAs in sink tissue in grafting experiments. However, we conclude that profiling phloem components combined to grafting studies is still not sufficient to doubtless decide whether a phloem-translocatable macromolecule is really a long-distance signal or not.

## Methods

### Plant material and growth conditions

For hydroponic growth, *Brassica napus *(cv. Drakkar, Serasem GIE, la Chapelle d'Armentiers, France) seeds were germinated on wet filter paper for 1 week. Germ buds were transferred to plastic boxes containing nutrient medium for 10 weeks. Nutrient medium: 0.6 mM NH_4_NO_3_, 1 mM Ca(NO_3_)_2_*4H_2_O, 0.04 mM Fe-EDTA, 0.5 mM K_2_HPO_4_, 0.5 mM K_2_SO_4_, 0.4 mM Mg(NO_3_)_2_*6H_2_O. Micro nutrients added: 0.8 μM ZnSO_4_*7H_2_O, 9 μM MnCl_2_*4H_2_O, 0.1 μM Na_2_MoO_4_*2H_2_O, 23 μM H_3_BO_3_, 0.3 μM CuSO_4_*5H_2_O. The pH was adjusted to 4.7 with 37% HCl. Nutrient solutions were changed after 4 weeks, and then renewed once a week. After 5 to 6 weeks, media were constantly aerated by an aquarium air pump (Sera, Heinsberg). Sulfur and copper starvation were applied for two, and iron starvation for three weeks before flowering started by changing to medium without sulfur, copper, or iron, respectively. Here, 0.5 mM K_2_SO_4 _were substituted by 0.5 mM K_2_HPO_4 _and instead of ZnSO_4_*7H_2_O and CuSO_4_*5H_2_O as micro nutrients, 1 μM ZnCl_2 _and 1 μM CuCl_2_*2H_2_O were added for low sulfate experiments. For copper deprivation, the 0.3 μM CuSO_4_*5H_2_O were omitted from the full nutrient solution. For low iron experiments Fe-EDTA was omitted from the medium.

For the growth of *Arabidopsis thaliana *WT (ecotype Ler-0) and *hen1-1 *[47] mutant plant seeds (NASC code N6583) were surface-sterilized in 70% (v/v) ethanol for 3 min and further incubated in 20% sodium hypochlorite solution containing 0.1% (v/v) surfactant (Triton X-100) for 10 min. After exhaustive washing with sterile water, seeds were placed on plates on half-concentrated MS medium [48] supplemented with 1% (w/v) sucrose and solidified with 0.7% (w/v) agar. After keeping them in the dark for three days at 4°C, seeds were germinated by transferring the plates in a growth chamber under controlled long day conditions (16 h day, 8 h night) at 25°C for 13 days. For hydroponic cultivation these plantlets were transferred into plastic boxes containing the nutrient solution previously described in [49] with minor modifications in the content of magnesium sulfate, boric acid and potassium dihydrogen phosphate (4 mM MgSO_4_*7H_2_O and 0.1 mM H_3_BO_3_, 2.5 mM KH_2_PO_4_). The hydroponic growth was carried out under short day conditions (8 h day at 20°C, 16 h night at 16°C). For sulfur deprivation experiments starvation was applied directly after the transfer of plantlets to hydroponic culture with nutrient solution omitting all sulfate-containing components for two weeks. Instead of MgSO_4_*7H_2_O 0.8 mM MgCl_2_*6H_2_O were added to the medium. Phosphate starvation was performed analogously in nutrient solution that contained potassium nitrate instead of potassium dihydrogen phosphate.

### Micrografting experiments

For micrografting experiments four-day-old *Arabidopsis thaliana *wild type and *hen1-1 *mutant seedlings were cut transversely using a sterile small razor blade part and combined within silicon tubing (0.3 mm internal diameter) as previously described [50]. The grafts were grown on 1.5% (w/v) agar plates with half-strength MS medium for nine days under controlled short day conditions. Successfully grafted plantlets were subsequently grown hydroponically for two weeks before plant material from stock and scion was harvested. To avoid contaminations, the area close to the graft union was omitted from sampling and grafts were microscopically inspected for adventitious root formation, what led to exclusion from analysis.

### Sampling and RNA isolation

Phloem sampling from *Brassica napus *plants was performed as described earlier [1,12] from 4 - 8 small punctures into the inflorescence stems. After discarding the first droplets to avoid contaminations, 500 μl to 1.5 ml phloem sap from three independent sets of plants were obtained, yielding about 10-50 μg of total RNA. Total RNA from phloem sap was isolated by Trizol LS reagent (Invitrogen) according to manufacturer's instructions.

RNA from 100 mg frozen material of stem, leaf and root tissue of *Brassica napus *and *Arabidopsis thaliana*, respectively, was extracted using the normal Trizol reagent. Total RNA from all samples was dissolved in 25 μl DEPC-treated water and RNA concentrations were determined photometrically with a Biophotometer (Eppendorf).

### Microarray hybridization

Microarray assays were performed by LC Sciences (Houston, Texas). The assays started from 2 to 5 μg total RNA samples that were size fractionated using a YM-100 Microcon centrifugal filter (Millipore) and the sRNAs (< 300 nt) isolated were 3'-extended with a poly(A) tail using poly(A) polymerase. An oligonucleotide tag was then ligated to the poly(A) tail for later fluorescent dye staining. Two different tags were used for the two RNA samples in dual-sample experiments. Hybridization was performed overnight on μParaflo microfluidic chips using a micro-circulation pump (Atactic Technologies). On the commercial microfluidic chip, each detection probe consisted of a chemically modified nucleotide coding segment complementary to a known target plant miRNA (from miRBase, http://microrna.sanger.ac.uk/sequences/, releases 10.0 (-S), 10.1(-Fe) or 11.0 (-Cu)). The known plant miRNAs were mainly from *Arabidopsis thaliana*, *Oryza sativa*, *Populus trichocarpa *and *Physcomitrella patens*. Among the total number of unique miRNA sequences (release 10.0, 623 miRNAs, 10.1, 653 miRNAs and 11.0, 714 miRNAs) all arrays contained a constant number of 154 miRNAs from *Arabidopsis thaliana*. Additionally to these known miRNAs, the customized array contained a set of 85 sRNAs of unknown function that were derived from an earlier high-throughput sequencing experiment of phloem sap [1] (sequences and accession numbers in additional file 5). Coding segments were coupled to a spacer segment of polyethylene glycol to place the coding segment away from the substrate. The detection probes were prepared by *in situ *synthesis using PGR (photogenerated reagent) chemistry. The hybridization melting temperatures were balanced by chemical modifications of the detection probes. For hybridization 100 μL 6 × SSPE buffer (0.90 M NaCl, 60 mM Na_2_HPO_4_, 6 mM EDTA, pH 6.8) containing 25% formamide at 34°C were used. After hybridization, signals were detected after fluorescence labeling using tag-specific Cy3 and Cy5 dyes. Hybridization images were collected using a laser scanner (GenePix 4000B, Molecular Devices) and digitized using Array-Pro image analysis software (Media Cybernetics). Data were analyzed by first subtracting the background and then normalizing the signals using a LOWESS (locally-weighted regression) filter.

To allow inter-array comparisons of FN samples, signal intensities were normalized to the median signal intensity of each sample and p-values of the t-test were calculated for the three replicates of each organ (phloem, leaves, and roots). Signals with p-values lower than 0.05 were regarded as being differential.

For the stress experiments (two color hybridizations), the ratio of the two sets of detected signals (log2 transformed, balanced) and p-values of the t-test were calculated and signals with p-values lower than 0.01 were regarded as being differential. Since only one array per stress was hybridized, we further restricted the data evaluation to sRNAs that showed a signal intensity of >100 in the FN or the stressed sample, an accumulation upon stress, and a more than four-fold difference (log2s of >2 or <-2) between stress and FN. All microarray data have been submitted to GEO, series accession No. GSE20263.

### Semi-quantitative RT-PCR

For semi-quantitative RT-PCR (sqRT-PCR), Trizol isolated RNA was cleaned with the RNeasy Plant Mini Kit (Qiagen) and a DNase I digest following the manufacturers instructions was performed. For nutrient stress-responsive marker gene and miRNA target transcript analysis, 500 - 1000 ng RNA were used for cDNA synthesis in the presence of 2.5 μM oligo(dT)_20 _primer (Qiagen), 0.5 mM dNTPs, 5 mM DTT (Invitrogen), 40 U RNaseOUT RNase Inhibitor (Invitrogen) and 200 U M-MLV reverse trancriptase (Promega) in 1× M-MLV reverse transcriptase reaction buffer (Promega) in a final volume of 20 μl. The reverse transcription reactions were carried out in a Primus Thermocycler (Peqlab) at 50°C for 45 min followed by 70°C for 15 min to denature the reverse transcriptase enzyme. 2 μl of the reverse transcription reaction were used for each PCR amplification with gene specific oligonucleotide primer pairs (additional file 6). The reaction mixtures containing 1.5 mM MgCl_2 _(Invitrogen), 0.2 mM dNTPs (Promega), 0.2 μM of both forward and backward primer and 2 U of Paq5000 DNA Polymerase in a 50 μl volume of 1× Paq5000 DNA polymerase buffer (Agilent Technologies) were divided into three equal volumes in reaction tubes and semi-quantitative RT-PCR was performed with different cycle numbers under the following conditions: 30 s at 94°C, 30 s at 55°C, 1 min at 72°C and a 10 min end-elongation step at 72°C. The PCR reaction was stopped after a certain number of cycles and PCR products were separated electrophoretically in 2% (w/v) agarose gels for size estimation and semi-quantitative analysis.

PCR of mature miRNAs was performed by following the method of Shi and Chiang [51]. Total RNA (1 μg) was first polyadenylated by a poly(A) polymerase [*E*-PAP, Poly(A) Tailing Kit (Ambion)] at 37°C for 1 h in a 50-μL reaction mixture containing 1× *E*-PAP buffer, 2.5 mM MnCl_2_, 1 mM ATP and 1 U *E*-PAP. Samples were purified from *E*-PAP by a further RNA extraction using TriFast FL reagent (Peqlab) and resolved in 50 μl DEPC-treated water. 10 μl of the polyadenylated RNA samples were used as a template for reverse transcription performed as described above using 0.5 μg poly(T) adapter instead of the oligo(dT)_20 _primer. miRNAs were subsequently amplified using 1 μl of the reverse transcribed sample, miRNA-specific forward and poly(T) adapter-specific reverse primers (additional file 6) under the same PCR-cycler conditions used in sqRT-PCR described above.

### RNA gel blot analysis

Gel blot analyzes were performed on 15% denaturing urea gels as described earlier [1,52].

## Authors' contributions

AB and FS carried out the plant growth, stress and microarray experiments. AB was also involved in microarray data analysis and evaluation. JP carried out the micrografting experiments, miRNA and target analyses. AB and JP drafted the manuscript. JK conceived of the study, participated in its design, coordination, data analysis, and drafted the manuscript. All authors read and approved the final manuscript.

## Supplementary Material

Additional file 1 **Comparison of miRNA abundance in phloem sap vs. inflorescence stem**. Comparison of sRNA microarray analysis of stem tissue (green) and phloem sap (blue) of *Brassica napus*. Only known miRNAs present on the commercial array, only one member per family are depicted. The upper graphs show the signal intensities on the array while the lower depict the log2 differences between phloem and inflorescence stem. Insets show RNA gel blot analyses of selected miRNAs from an independent experiment. Numbers indicate the number of sequences that were previously obtained by phloem sap sequencing [1], asterisks (*) indicate sequences from miRNA stars. Click here for file

Additional file 2 **Comparison of sRNA abundances in phloem, leaves and roots**. sRNA microarray comparison of phloem (blue), leaf (green) and root (red) tissue of *Brassica napus *plants from biologically independent replications (n = 3). To allow inter-array comparison, signal intensities were normalized to the median signal of each sample. Only known miRNAs present on the commercial array and only one member per family are depicted. Click here for file

Additional file 3 **Transcript analysis of known nutrient stress-specific genes**. Transcript analysis of known nutrient stress-specific genes in leaf and root tissue of hydroponically grown *Brassica napus *plants by semi-quantitative RT-PCR after 25, 30 and 35 cycles under -S, -Cu and -Fe compared to full nutrition (FN). Click here for file

Additional file 4 **Accumulation of three miR395 targets in WT and hen1-1 shoots grown under full nutrition**. Levels of the targets SULTR2;1, APS1 and APS4 in shoots as detected by sqRT-PCR (35 cycles, UBC10, At5g53300 served as a control). FN: full nutrition. Click here for file

Additional file 5 **Sequences of the unknown phloem sap sRNAs represented on the microarrays**. Phloem sap small RNA sequences of *Brassica napus *(Bn_PsRNAs) that were contained on the sRNA microarray (sequences were derived from high-throughput sequencing of *B. napus *phloem sap published in [1]). Click here for file

Additional file 6 **List of oligonucleotides used**. Oligonucleotide sequences used for the detection of nutrient stress-specific miRNAs by RNA gel blots or by semi-quantitative RT-PCR, for the analysis of miR395 target genes, and for transcript detection of nutrient-responsive genes by semi-quantitative RT-PCR. Click here for file

## References

[B1] Buhtz A Springer F Chappell L Baulcombe DC Kehr J Identification and characterization of small RNAs from the phloem of *Brassica napus* Plant J 2008 53 739 749 10.1111/j.1365-313X.2007.03368.x 18005229

[B2] Pant BD Buhtz A Kehr J Scheible WR MicroRNA399 is a long-distance signal for the regulation of plant phosphate homeostasis Plant J 2008 53 731 738 10.1111/j.1365-313X.2007.03363.x 17988220 PMC2268993

[B3] Lin SI Chiang SF Lin WY Chen JW Tseng CY Wu PC Chiou TJ Regulatory network of microRNA399 and PHO2 by systemic signaling Plant Physiol 2008 147 732 746 10.1104/pp.108.116269 18390805 PMC2409027

[B4] Fujii H Chiou TJ Lin SI Aung K Zhu JK A miRNA involved in phosphate-starvation response in *Arabidopsis* Curr Biol 2005 15 2038 2043 10.1016/j.cub.2005.10.016 16303564

[B5] Bari R Pant BD Stitt M Scheible W PHO2, microRNA399, and PHR1 define a phosphate-signaling pathway in plants Plant Physiol 2006 141 988 999 10.1104/pp.106.079707 16679424 PMC1489890

[B6] Chiou T Aung K Lin S Wu C Chiang S Su C Regulation of phosphate homeostasis by microRNA in *Arabidopsis* Plant Cell 2006 18 412 421 10.1105/tpc.105.038943 16387831 PMC1356548

[B7] Aung K Lin S Wu C Huang Y Su C Chiou T pho2, a phosphate overaccumulator, is caused by a nonsense mutation in a microRNA399 target gene Plant Physiol 2006 141 1000 1011 10.1104/pp.106.078063 16679417 PMC1489903

[B8] Jones-Rhoades MW Bartel DP Computational identification of plant microRNAs and their targets, including a stress-induced miRNA Mol Cell 2004 14 787 799 10.1016/j.molcel.2004.05.027 15200956

[B9] Sunkar R Chinnusamy V Zhu J Zhu J Small RNAs as big players in plant abiotic stress responses and nutrient deprivation Trends Plant Sci 2007 12 301 309 10.1016/j.tplants.2007.05.001 17573231

[B10] Yamasaki H Abdel-Ghany SE Cohu CM Kobayashi Y Shikanai T Pilon M Regulation of copper homeostasis by microRNA in *Arabidopsis* J Biol Chem 2007 282 16369 16378 10.1074/jbc.M700138200 17405879

[B11] Abdel-Ghany SE Pilon M MicroRNA-mediated systemic down-regulation of copper protein expression in response to low copper availability in *Arabidopsis* J Biochem 2008 283 15932 15945 10.1074/jbc.M801406200 PMC3259626 18408011

[B12] Giavalisco P Kapitza K Kolasa A Buhtz A Kehr J Towards the proteome of *Brassica napus *phloem sap Proteomics 2006 6 896 909 10.1002/pmic.200500155 16400686

[B13] Varkonyi-Gasic E Wu R Wood M Walton EF Hellens RP A highly sensitive RT-PCR method for detection and quantification of microRNAs Plant Methods 2007 3 12 10.1186/1746-4811-3-12 17931426 PMC2225395

[B14] Pant BD Musialak-Lange M Nuc P May P Buhtz A Kehr J Walther D Scheible W Identification of nutrient-responsive Arabidopsis and rapeseed microRNAs by comprehensive real-time polymerase chain reaction profiling and small RNA sequencing Plant Physiol 2009 150 1541 1555 10.1104/pp.109.139139 19465578 PMC2705054

[B15] Jones-Rhoades MW Bartel DP Bartel B MicroRNAs and their regulatory roles in plants Ann Rev Plant Biol 2006 57 19 53 10.1146/annurev.arplant.57.032905.105218 16669754

[B16] Yao Y Guo G Ni Z Sunkar R Du J Zhu J Sun Q Cloning and characterization of microRNAs from wheat (*Triticum aestivum *L.) Genome Biol 2007 8 R96 10.1186/gb-2007-8-6-r96 17543110 PMC2394755

[B17] Unver T Budak H Conserved microRNAs and their targets in model grass species *Brachypodium distachyon* Planta 2009 230 659 669 10.1007/s00425-009-0974-7 19585143

[B18] Liang R Li W Li Y Tan C Li J Jin Y Ruan K An oligonucleotide microarray for microRNA expression analysis based on labeling RNA with quantum dot and nanogold probe Nucl Acids Res 2005 33 e17 10.1093/nar/gni019 15684409 PMC548377

[B19] Boualem A Laporte P Jovanovic M Laffont C Plet J Combier J Niebel A Crespi M Frugier F microRNA166 controls root and nodule development in *Medicago truncatula* Plant J 2008 54 876 887 10.1111/j.1365-313X.2008.03448.x 18298674

[B20] Axtell MJ Bartel DP Antiquity of microRNAs and their targets in land plants Plant Cell 2005 17 1658 1673 10.1105/tpc.105.032185 15849273 PMC1143068

[B21] Wang J Wang L Mao Y Cai W Xue H Chen X Control of root cap formation by microRNA-targeted auxin response factors in *Arabidopsis* Plant Cell 2005 17 2204 2216 10.1105/tpc.105.033076 16006581 PMC1182483

[B22] Yang T Xue L An L Functional diversity of miRNA in plants Plant Sci 2007 172 423 432 10.1016/j.plantsci.2006.10.009

[B23] Rajagopalan R Vaucheret H Trejo J Bartel DP A diverse and evolutionarily fluid set of microRNAs in *Arabidopsis thaliana* Genes Dev 2006 20 3407 3425 10.1101/gad.1476406 17182867 PMC1698448

[B24] Xie Z Allen E Fahlgren N Calamar A Givan SA Carrington JC Expression of Arabidopsis MIRNA genes Plant Physiol 2005 138 2145 2154 10.1104/pp.105.062943 16040653 PMC1183402

[B25] Meyers BC Axtell MJ Bartel B Bartel DP Baulcombe D Bowman JL Cao X Carrington JC Chen X Green PJ Griffiths-Jones S Jacobsen SE Mallory AC Martienssen RA Poethig RS Qi Y Vaucheret H Voinnet O Watanabe Y Weigel D Zhu JK Criteria for annotation of plant microRNAs Plant Cell 2008 20 3186 3190 10.1105/tpc.108.064311 19074682 PMC2630443

[B26] Yamasaki H Hayashi M Fukazawa M Kobayashi Y Shikanai T *SQUAMOSA *promoter binding protein-like7 is a central regulator for copper homeostasis in *Arabidopsis* Plant Cell 2009 21 347 361 10.1105/tpc.108.060137 19122104 PMC2648088

[B27] Song C Fang J Li X Liu H Chao CT Identification and characterization of 27 conserved microRNAs in citrus Planta 2009 230 671 685 10.1007/s00425-009-0971-x 19585144 PMC2729984

[B28] Schwab R Palatnik JF Riester M Schommer C Schmid M Weigel D Specific effects of microRNAs on the plant transcriptome Dev Cell 2005 8 517 527 10.1016/j.devcel.2005.01.018 15809034

[B29] Barker AV Pilbeam DJ Handbook of plant nutrition 2007 Boca Raton: CRC Press

[B30] Chen Y Shi J Tian G Zheng S Lin Q Fe deficiency induces Cu uptake and accumulation in *Commelina communis* Plant Sci 2004 166 1371 1377 10.1016/j.plantsci.2004.01.018

[B31] Puig S Andrés-Colás N García-Molina A Penarrubia L Copper and iron homeostasis in Arabidopsis: responses to metal deficiencies, interactions and biotechnological applications Plant Cell Environ 2007 30 271 290 10.1111/j.1365-3040.2007.01642.x 17263774

[B32] Jeong J Guerinot ML Homing in on iron homeostasis in plants Trends Plant Sci 2009 14 280 285 10.1016/j.tplants.2009.02.006 19375375

[B33] Svistoonoff S Creff A Reymond M Sigoillot-Claude C Ricaud L Blanchet A Nussaume L Desnos T Root tip contact with low-phosphate media reprograms plant root architecture Nat Genet 2007 39 792 796 10.1038/ng2041 17496893

[B34] Ward JT Lahner B Yakubova E Salt DE Kashchandra GR The effect of iron on the primary root elongation of Arabidopsis during phosphate deficiency Plant Physiol 2008 147 1181 1191 10.1104/pp.108.118562 18467463 PMC2442553

[B35] La Fontaine S Quinn JM Nakamoto SS Page MD Goehre V Moseley JL Kropat J Merchant S Copper-dependent iron assimilation pathway in the model photosynthetic eukaryote *Chlamydomonas reinhardtii* Eukaryot Cell 2002 1 736 757 10.1128/EC.1.5.736-757.2002 12455693 PMC126744

[B36] Pilon M Abdel-Ghany SE Cohu CM Gogolin KA Ye H Copper cofactor delivery in plant cells Curr Opin Plant Biol 2006 9 256 263 10.1016/j.pbi.2006.03.007 16616609

[B37] Park W Li J Song R Messing J Chen X CARPEL FACTORY, a Dicer homolog, and HEN1, a novel protein, act in microRNA metabolism in *Arabidopsis thaliana* Curr Biol 2002 12 1484 1495 10.1016/S0960-9822(02)01017-5 12225663 PMC5137372

[B38] Yoo B Kragler F Varkonyi-Gasic E Haywood V Archer-Evans S Lee YM Lough TJ Lucas WJ A systemic small RNA signaling system in plants Plant Cell 2004 16 1979 2000 10.1105/tpc.104.023614 15258266 PMC519190

[B39] Kawashima CG Yoshimoto N Maruyama-Nakashita A Tsuchiya YN Saito K Takahashi H Dalmay T Sulphur starvation induces the expression of microRNA-395 and one of its target genes but in different cell types Plant J 2009 57 313 321 10.1111/j.1365-313X.2008.03690.x 18801012

[B40] Noji M Goulart Kawashima C Obayashi T Saito K *In silico *assessment of gene function involved in cysteine biosynthesis in Arabidopsis: expression analysis of multiple isoforms of serine acetyltransferase Ami 2006 30 163 171 10.1007/s00726-005-0253-2 16525754

[B41] Dunoyer P Himber C Ruiz-Ferrer V Alioua A Voinnet O Intra- and intercellular RNA interference in *Arabidopsis thaliana *requires components of the microRNA and heterochromatic silencing pathways Nat Genet 2007 39 848 856 10.1038/ng2081 17558406

[B42] Parizotto EA Dunoyer P Rahm N Himber C Voinnet O In vivo investigation of the transcription, processing, endonucleolytic activity, and functional relevance of the spatial distribution of a plant miRNA Genes Dev 2004 18 2237 2242 10.1101/gad.307804 15371337 PMC517516

[B43] Nogueira F Chitwood D Madi S Kazuhiro O Schnable P Scalon M Timmermans MC Regulation of small RNA accumulation in the maize shoot apex PLoS Genet 2009 5 e1000320 10.1371/journal.pgen.1000320 19119413 PMC2602737

[B44] Alvarez JP Pekker I Goldshmidt A Blum E Amsellem Z Eshed Y Endogenous and synthetic microRNAs stimulate simultaneous, efficient, and localized regulation of multiple targets in diverse species Plant Cell 2006 18 1134 10.1105/tpc.105.040725 16603651 PMC1456869

[B45] Valoczi A Varallyay E Kauppinen S Burgyan J Havelda Z Spatio-temporal accumulation of microRNAs is highly coordinated in developing plant tissues Plant J 2006 47 140 151 10.1111/j.1365-313X.2006.02766.x 16824182

[B46] Oparka KJ Cruz SS The great escape: phloem transport and unloading of macromolecules Ann Rev Plant Biol Plant Mol Biol 2000 51 323 347 10.1146/annurev.arplant.51.1.323 15012195

[B47] Chen X Liu J Cheng Y Jia D HEN1 functions pleiotropically in Arabidopsis development and acts in C function in the flower Development 2002 129 1085 1094 10.1242/dev.00114 11874905 PMC5137379

[B48] Murashige T Skoog F A revised medium for rapid growth and bioassays with tobacco tissue cultures Physiol Plant 1962 15 473 497 10.1111/j.1399-3054.1962.tb08052.x

[B49] Gibeaut DM Hulett J Cramer GR Seemann JR Maximal biomass of *Arabidopsis thaliana *using a simple, low-maintenance hydroponic method and favorable environmental conditions Plant Physiol 1997 115 317 319 10.1104/pp.115.2.317 9342857 PMC158488

[B50] Turnbull CGN Booker JP Leyser HMO Micrografting techniques for testing long-distance signalling in *Arabidopsis* Plant J 2002 32 255 262 10.1046/j.1365-313X.2002.01419.x 12383090

[B51] Shi R Chiang VL Facile means for quantifying microRNA expression by real-time PCR BioTechniques 2005 39 519 525 10.2144/000112010 16235564

[B52] Chappell L Baulcombe DC Molnár A Coico R, Kowalik T, Quarles JM, Stevenson B, Taylor RK, Simon AE, Downey T Isolation and cloning of small RNAs from virus-infected plants Current Protocols in Microbiology 2005 Hoboken, N.J.: John Wiley & Sons 16H.2.1 16H.2.17 10.1002/9780471729259.mc16h02s00 18770585

