# Supplementary material for: Phloem small RNAs, nutrient stress responses, and systemic mobility
Source: BMC Plant Biol. 2010 Apr 13;10:64. doi: 10.1186/1471-2229-10-64 (PMC2923538; doi:10.1186/1471-2229-10-64)
Supplement: Additional file 1 — Comparison of miRNA abundance in phloem sap vs. inflorescence stem. Comparison of sRNA microarray analysis of stem tissue (green) and phloem sap (blue) of Brassica napus. Only known miRNAs present on the commercial array, only one member per family are depicted. The upper graphs show the signal intensities on the array while the lower depict the log2 differences between phloem and inflorescence stem. Insets show RNA gel blot analyses of selected miRNAs from an independent experiment. Numbers indicate the number of sequences that were previously obtained by phloem sap sequencing [1], asterisks (*) indicate sequences from miRNA stars. [file 1471-2229-10-64-S1.PPT]

## Slide 1
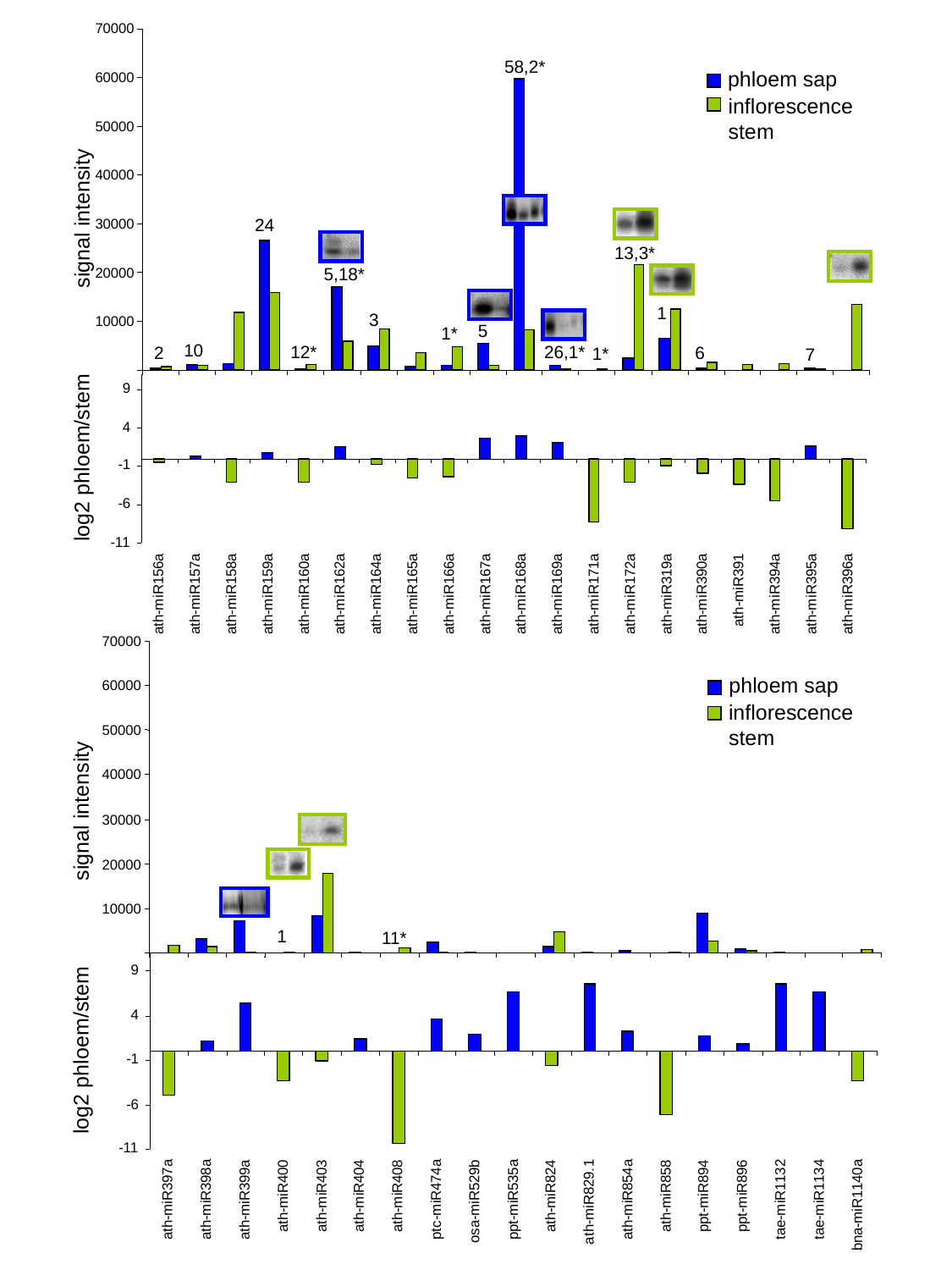

70000
58,2*
phloem sap
60000
inflorescence stem
50000
40000
signal intensity
24
30000
13,3*
5,18*
20000
1
3
10000
5
1*
10
12*
26,1*
2
6
1*
7
log2 phloem/stem
70000
phloem sap
60000
inflorescence stem
50000
40000
signal intensity
30000
20000
10000
1
11*
log2 phloem/stem
