# Supplementary figures and images for: Phloem small RNAs, nutrient stress responses, and systemic mobility
Source: BMC Plant Biol. 2010 Apr 13;10:64. doi: 10.1186/1471-2229-10-64 (PMC2923538; doi:10.1186/1471-2229-10-64)

## Slide 1
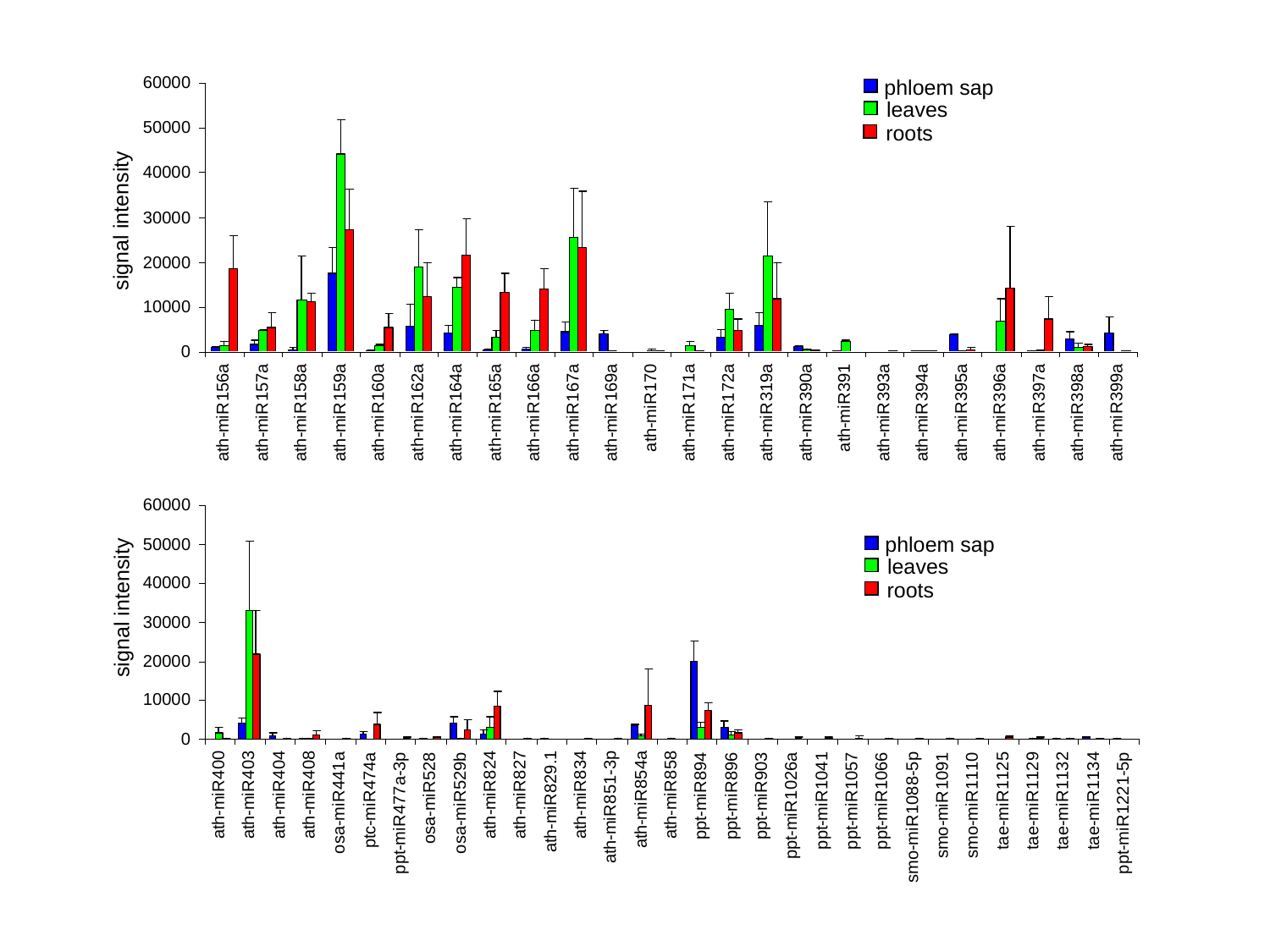

phloem sap
leaves
roots
signal intensity
phloem sap
leaves
roots
signal intensity

Supplement: Additional file 2 — Comparison of sRNA abundances in phloem, leaves and roots. sRNA microarray comparison of phloem (blue), leaf (green) and root (red) tissue of Brassica napus plants from biologically independent replications (n = 3). To allow inter-array comparison, signal intensities were normalized to the median signal of each sample. Only known miRNAs present on the commercial array and only one member per family are depicted. [file 1471-2229-10-64-S2.PPT]

## Slide 1
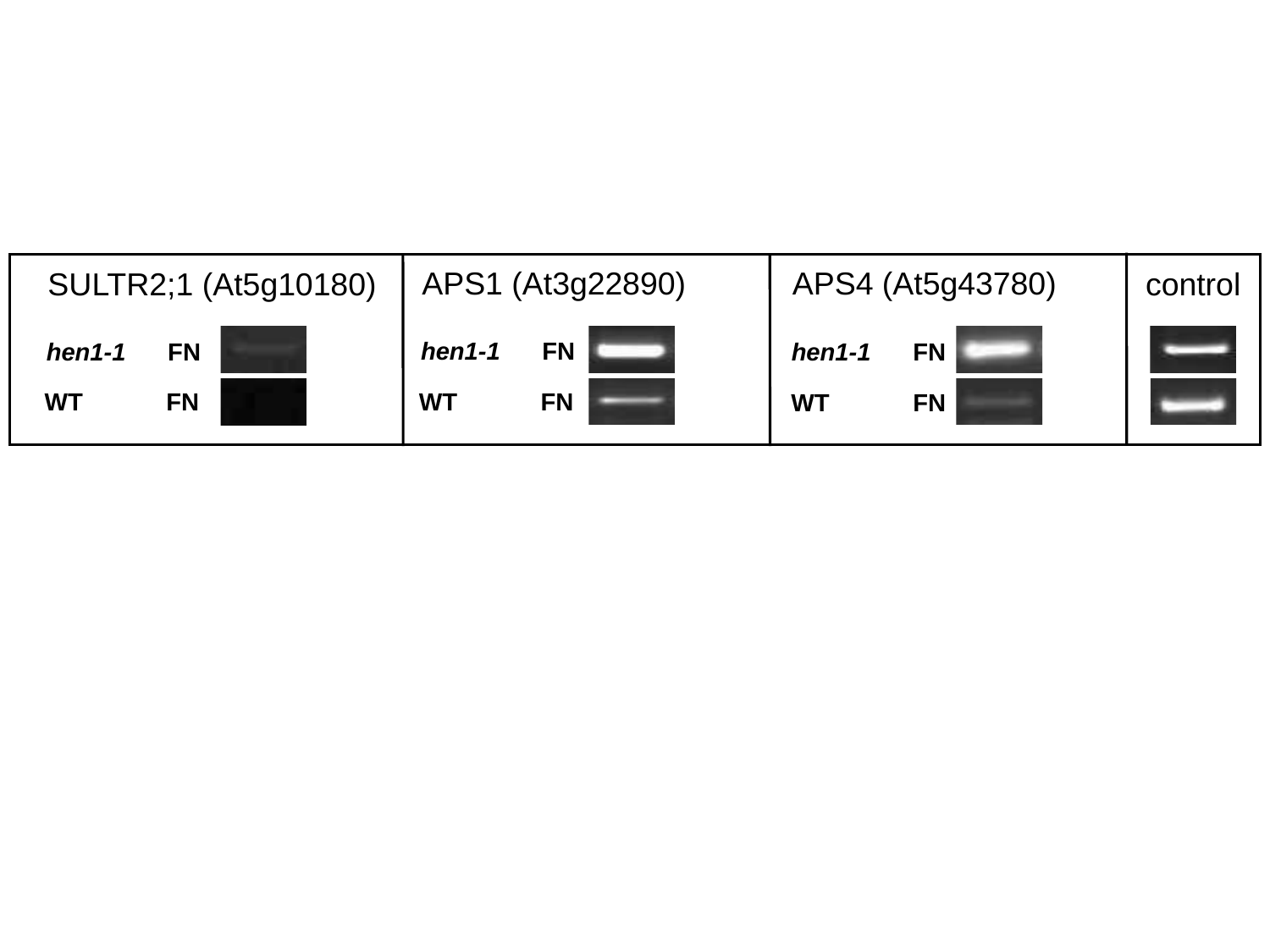

APS1 (At3g22890)
APS4 (At5g43780)
SULTR2;1 (At5g10180)
control
hen1-1 FN
hen1-1 FN
hen1-1 FN
WT FN
WT FN
WT FN

Supplement: Additional file 4 — Accumulation of three miR395 targets in WT and hen1-1 shoots grown under full nutrition. Levels of the targets SULTR2;1, APS1 and APS4 in shoots as detected by sqRT-PCR (35 cycles, UBC10, At5g53300 served as a control). FN: full nutrition. [file 1471-2229-10-64-S4.PPT]
