# Supplementary material for: Phloem small RNAs, nutrient stress responses, and systemic mobility
Source: BMC Plant Biol. 2010 Apr 13;10:64. doi: 10.1186/1471-2229-10-64 (PMC2923538; doi:10.1186/1471-2229-10-64)
Supplement: Additional file 3 — Transcript analysis of known nutrient stress-specific genes. Transcript analysis of known nutrient stress-specific genes in leaf and root tissue of hydroponically grown Brassica napus plants by semi-quantitative RT-PCR after 25, 30 and 35 cycles under -S, -Cu and -Fe compared to full nutrition (FN). [file 1471-2229-10-64-S3.PPT]

## Slide 1
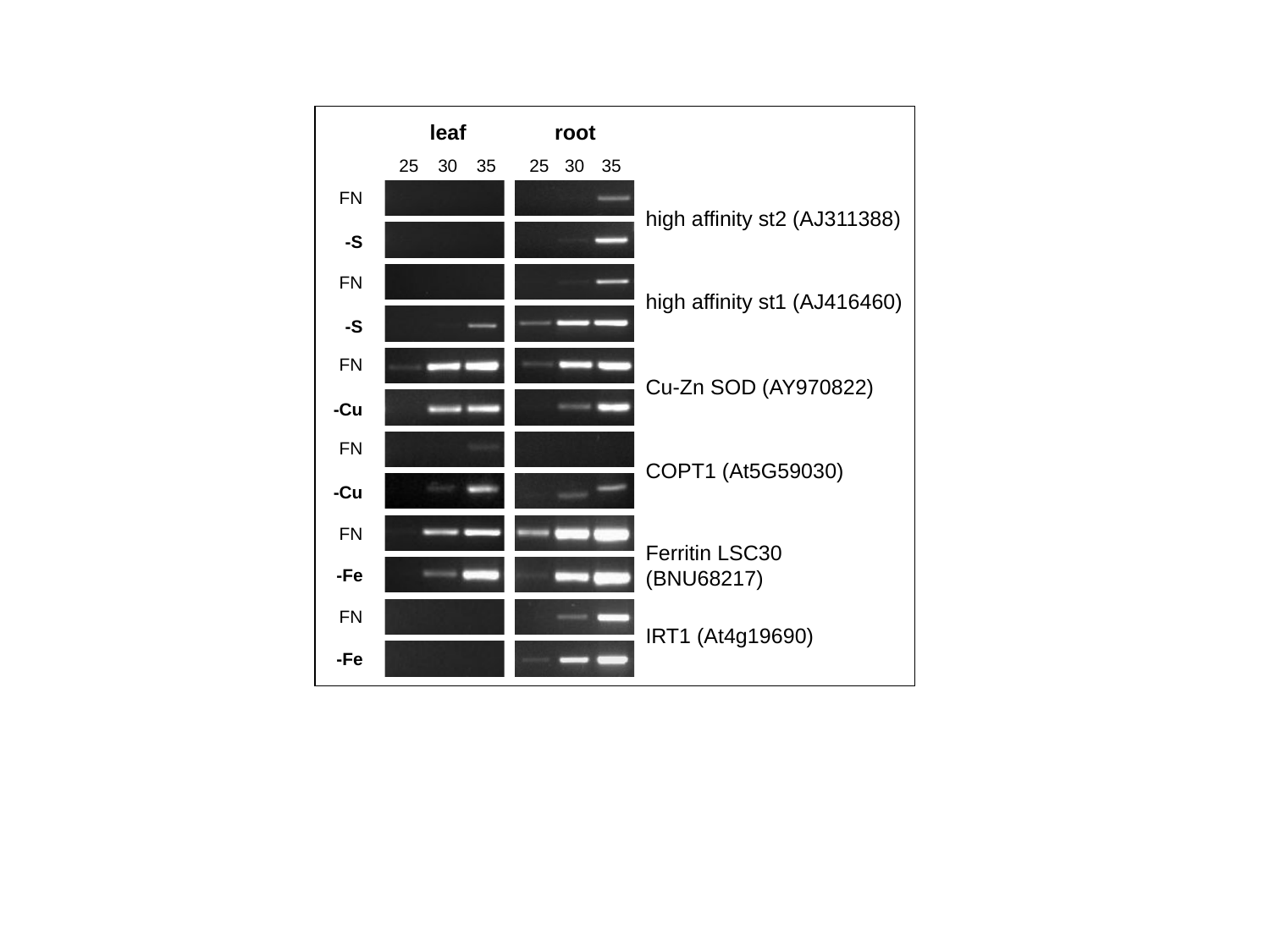

leaf
root
25
30
35
25
30
35
FN
high affinity st2 (AJ311388)
-S
FN
high affinity st1 (AJ416460)
-S
FN
Cu-Zn SOD (AY970822)
-Cu
FN
COPT1 (At5G59030)
-Cu
FN
Ferritin LSC30 (BNU68217)
-Fe
FN
IRT1 (At4g19690)
-Fe
